# Genomic Diversity and Evolutionary Insights of Avian Paramyxovirus-1 in Avian Populations in Pakistan

**DOI:** 10.3390/v16091414

**Published:** 2024-09-05

**Authors:** Muhammad Zubair Shabbir, Sahar Mahmood, Aziz Ul-Rahman, Ashley C. Banyard, Craig S. Ross

**Affiliations:** 1 Institute of Microbiology, University of Veterinary and Animal Sciences, Lahore 54000, Pakistan; shabbirmz@uvas.edu.pk; 2 Virology Department, Animal and Plant Health Agency (APHA), Addlestone KT15 3NB, UKashley.banyard@apha.gov.uk (A.C.B.); 3 Department of Pathobiology and Biomedical Sciences, MNS University of Agriculture, Multan 66000, Pakistan; 4 WOAH/FAO International Reference Laboratory for Avian Influenza, Swine Influenza and Newcastle Disease, Animal and Plant Health Agency (APHA), Addlestone KT15 3NB, UK

**Keywords:** avian paramyxovirus-1, evolutionary dynamics, genotypes and sub-genotypes, Pakistan

## Abstract

The virulent form of Avian paramyxovirus-1 (APMV-1), commonly known as Newcastle Disease Virus (NDV), is a pathogen with global implications for avian health, affecting both wild and domestic bird populations. In Pakistan, recurrent Newcastle Disease (caused by NDV) outbreaks have posed significant challenges to the poultry industry. Extensive surveillance in Pakistan over 20 years has demonstrated a dynamic genetic diversity among circulating APMV-1 strains, emphasizing the potential necessity for customized vaccination strategies and continuous surveillance. In this study, 13 APMV-1-positive isolates harboring four different APMV-1 genotypes circulating throughout Pakistan were identified. These included the highly virulent genotypes VII and XIII, genotype XXI, commonly associated with Columbiformes, and genotype II, hypothesized to have been detected following vaccination. These findings underscore the intricate interplay of mutational events and host-immune interactions shaping the evolving NDV landscape. This study advances our understanding of the evolutionary dynamics of APMV-1 in Pakistan, highlighting the need for tailored vaccination strategies and continuous surveillance to enable effective APMV-1 management in avian populations, further emphasizing the importance of globally coordinated strategies to tackle APMV-1, given its profound impact on wild and domestic birds.

## 1. Introduction

Avian Paramyxovirus-1 (APMV-1) is a member of the Avulavirinae subfamily within the Paramyxoviridae family. Virulent forms of APMV-1 (commonly termed Newcastle Disease Virus (NDV)) are the causative agent of Newcastle Disease (ND), which has a considerable impact on avian populations worldwide, both commercially and in the wild. Outbreaks of NDV have considerable impacts on domestic poultry production, and ND is endemic in large parts of the world. Even in resource-rich countries, outbreaks of NDV cause significant impacts that can be hard to manage and eradicate [1,2]. Consequently, NDV continues to play a pivotal role in shaping avian health, food security, and the economic stability of poultry producers worldwide.

The genome of APMV-1 comprises non-segmented, single-stranded, negative-sense RNA of approximately 15,192 nucleotides. This RNA encodes six structural proteins: nucleocapsid protein (NP), phosphoprotein (P), matrix protein (M), fusion protein (F), hemagglutinin-neuraminidase protein (HN), and large RNA-dependent RNA polymerase protein (L), plus two non-structural proteins, V and W, generated by RNA editing of the P gene [3]. The F and HN proteins are indispensable in viral attachment, fusion, and entry into host cells, as observed in other paramyxoviruses (reviewed in [4]), while NP, P, and L interact with the viral RNA to form the ribonucleoprotein complex required to replicate viral RNA [5].

Avian paramyxovirus-1 is present in two forms: virulent and avirulent strains. Strict guidance determines the declaration of Newcastle Disease, which follows guidelines set out by the World Organization for Animal Health (WOAH). Virulent forms (also, as declared by WOAH) must either have an Intracerebral Pathogenicity Index (ICPI) of >0.7 or a multi-basic cleavage site containing three or more lysine or arginine residues at position 112–116 of the F gene with a Phenylalanine present at position 117 [6]. Newcastle Disease is declared if a virulent form of APMV-1 is detected in poultry [6], and critically, detection of APMV-1s with a multi-basic cleavage site in non-poultry species does not constitute the presence of ND. Although APMV-1 infection can be defined as virulent or avirulent, significant differences in clinical signs are often seen, with avirulent APMV-1 often causing no clinical disease (avirulent) or low morbidity with zero mortality (termed lentogenic). In contrast, virulent forms can cause low mortality but with high morbidity, for example, increased weight loss and decreased egg production (termed mesogenic). Finally, the most virulent genotypes commonly result in high morbidity with high mortality (termed velogenic). The basis for this difference in clinical outcomes is not clear, although the major driver for virulence in chickens is the presence of the virulent cleavage site in the F gene [7,8], although other APMV-1 proteins and promoters have also been implicated in determinants of virulence [9,10,11,12].

Over the years, the effort to combat NDV infections in poultry populations in Pakistan has been marked by evolving challenges. Despite routine vaccination, the recurrence of outbreaks and the emergence of new genotypes highlight the intricate evolutionary nature of APMV-1 [13,14,15]. Research conducted in Pakistan has unveiled the changing genetic diversity and adaptability of circulating APMV-1 strains over time, indicating the distribution of various genotypes and highlighting the importance of tailored vaccination strategies, whether this is changes in vaccination regimes or targeted genotype-matched vaccines, and the need for continuous surveillance [13,14,16,17,18,19,20]. These processes collectively contribute to the perpetually evolving landscape of viral genetic diversity and host–pathogen relationships. Recent advancements have prompted the virus’s reclassification, comprehensively re-evaluated its genetic lineages, and provided fresh insights into virus diversity [21,22,23]. Furthermore, due to the co-circulation of multiple genotypes, designing effective vaccination strategies becomes a multifaceted task, given the genetic diversity that potentially undermines vaccine efficacy.

Here, we compare the detection and genetic composition of APMV-1 in Pakistan over the past two decades to establish evolutionary trends, genetic diversity, and prevalence of APMV-1 genotypes and, therefore, allow estimation of the temporal distribution of these genotypes in Pakistan.

## 2. Materials and Methods

### 2.1. Study Isolates

Thirteen APMV-1 isolates were recovered from multiple avian species from 2009 to 2021 (see Table 1). Depending upon clinical manifestation, a wide range of clinical specimens (brain, trachea, lung, spleen, proventriculus, and cecal tonsil) were collected from various bird types (chickens, parrots, pheasants, and pigeons) that were clinically suspected of having ND during field outbreaks and on-site visits at the diagnostic laboratory of the University of Veterinary and Animal Sciences (Lahore, Pakistan). Additionally, clinical material (oropharyngeal and cloacal swabs) from healthy green-winged teal (Anas carolinensis) was collected during the Avian Influenza surveillance program at the Indus River wetland sanctuary for virus isolation at the diagnostic laboratory (Table 1). Clinical samples from the field outbreaks and ducks were collected in separate cryovials containing 1.5 mL Brain Heart Infusion (BHI) medium with antimicrobials (Gentamicin 200 μg/mL, Penicillin 2000 IU/mL, and Fungizone 1.5 μg/mL). The cryovials were placed in coolers with ice packs, transported to the diagnostic laboratory, and stored at −80 °C until further processing. The clinical samples collected at the diagnostic laboratory were immediately placed in BHI medium and stored at −80 °C.

### 2.2. Virus Isolation (VI) and Confirmatory Analysis

Approximately 1–2 mL of homogenized tissue suspension of each host was filtered through a syringe filter (0.2 μm EMD Millipore Millex™, Milli-pore, Billerica, MA, USA), and the filtrate was then inoculated into 8–10-day-old embryonated chicken eggs (ECE) via chorioallantoic sac route per the protocol described previously [6]. The harvested allantoic fluid was first assessed for standard Hemagglutination assay [6] and later confirmed to be APMV-1 by reverse transcriptase-PCR-based amplification of the partial F gene using degenerate primers described previously [24].

### 2.3. Whole Genome Sequencing

Whole genome de novo sequencing of these thirteen isolates was conducted at the Animal and Plant Health Agency, UK, as described previously [25]. Briefly, the extracted vRNA was prepared for whole genome sequencing (WGS) by synthesizing complementary DNA (cDNA) using SuperScript IV (ThermoFisher Scientific, Paisley, UK), with double-stranded cDNA synthesized via a non-directional module (New England Biolabs, Hitchin, UK). The double-stranded cDNA was purified using AMPure beads (Beckman Coulter, High Wycombe, UK) for downstream library preparation, which was performed with a Nextera DNA Library Preparation Kit (Illumina, Cambridge, UK). The resultant library was sequenced using a NextSeq (Illumina, Cambridge, UK) instrument in line with the manufacturer’s instructions. *De novo* assembly was carried out using the paired-end reads obtained from the NextSeq (Illumina, Cambridge, UK) and were assembled using an in-house developed *de novo* assembly script (https://github.com/APHA-VGBR/WGS_Pipelines, accessed 8 January 2024). The complete genome sequences were submitted to the NCBI database and are accessible with the numbers OR367430-OR367442.

### 2.4. Genome Dataset Retrieval and Phylogenetic Analysis

The dataset of nucleotides for the F-gene sequences of the APMV-1 virus in Pakistan, in addition to sequences from various global locations representing the virus, were sourced from the NDV consortium (GitHub-NDVconsortium/NDV_Sequence_Datasets: Curated complete Fusion gene class I and class II sequence datasets) or from NCBI. Maximum-likelihood phylogenetic trees were inferred as described previously [26] using the full F-gene sequence (1662 bp) where available. Phylogenetic tree models were used: the General-Time reversal model for the pilot, genotype VII and genotype XIII trees, and Tamura-Nei for genotype XXI, as determined by ModelFinder [27]. Molecular clock estimations and mugration analysis were conducted using Treetime [28] individually for each genotype examined. Regions for analysis were determined by using the United Nations geoscheme [29]. Trees were visualized as previously described [30]. The estimated average evolutionary distances were calculated using MEGA X [31].

## 3. Results

### 3.1. Phylogenetic and Comparative Genome Analysis

**Figure 1:**
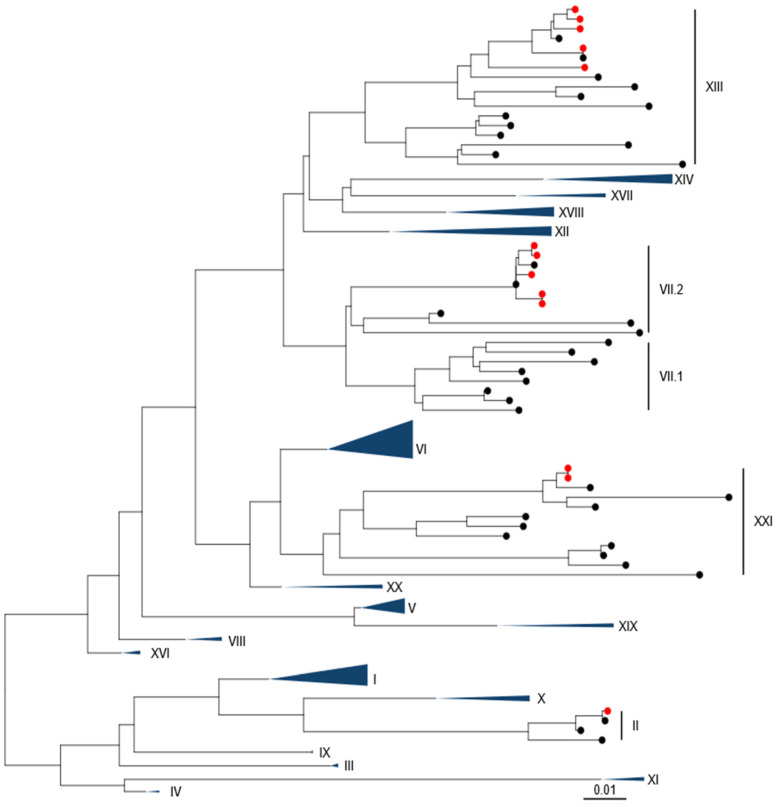
APMV-1 sequences obtained from Pakistan are demonstrated to be Genotype II, Genotype VII.2, Genotype XIII.2.1, and Genotype XXI.1.1. Maximum-likelihood phylogenetic tree of the F-gene from global, pre-defined APMV-1 sequences. Sequences are colored red for isolates from this study and black for all other sequences.

Phylogenetic analysis using pilot tree datasets demonstrated a single sequence was closely related to a genotype II strain (Figure 1 and Figure S1). Five of the sequences were closely related to two previous genotype VII.2 isolates. A further five isolates were found to be closely related to previous genotype XIII.2.1 sequences, while the remaining two isolates (from pigeons) were genotype XXI.1.2.

### 3.2. Phylogenetic Analysis of Genotype VII.2 APMV-1 Isolates

**Figure 2:**
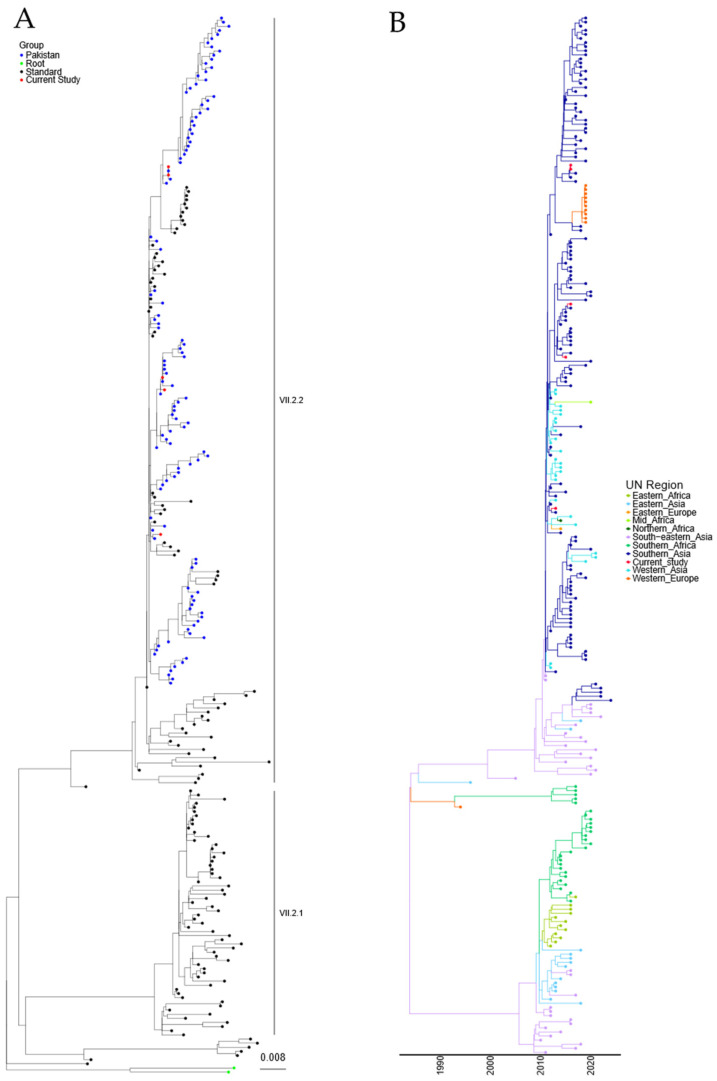
APMV-1 sequences designated genotype VII.2 are closely related to previous isolates observed in Pakistan. (**A**) Maximum-likelihood tree of the F-gene of five Pakistan isolates identified in this study, along with 251 F-gene sequences previously identified as genotype VII.2. Isolates from this study are colored red, previous Pakistan samples are colored blue, root sequences are green, and remaining samples are colored black. (**B**) Estimation of time-scaled phylogenetic and mugration analysis of genotype VII.2 isolates. Estimation of time-scaled phylogenetic analysis was conducted on the M-L tree to determine the likely dates of the last common ancestors for each isolate; subsequently, mugration analysis was conducted to determine the likely region of incursion. Regions are colored as stated.

Phylogenetic analysis of the sequences obtained from the five Pakistani genotype VII.2 strains was conducted using the 251 genotype VII.2 sequences deposited in the NDV consortium database and recent additions to the NCBI database, along with the two recommended root sequences [23]. A maximum-likelihood (ML) tree was generated (Figure 2A and Figure S2). Phylogenetic analysis further confirmed that the five sequenced APMV-1 isolates from Pakistan were genotype VII.2 and grouped closely with previously sequenced samples from Pakistan.

A time-resolved analysis was undertaken to estimate the date of the last common ancestor of the genotype VII.2 APMV-1 before introduction into Pakistan (first detection 2011), as well as the region of origin. Estimation of time-scaled phylogenetic analysis demonstrated that the last common ancestor before detection in Southern Asia was 12/2009 (lower and upper limit 07/2009–01/2010) for the initial incursion, based on the current data available (Figure 2B). Analysis of the region of initial incursion suggested that the incoming APMV-1 genotype VII.2 virus was from the Southeast Asian region (Figure 2B).

Both maximum-likelihood and time/region analysis of the Genotype VII.2 phylogenetic tree show that these viruses have split into at least two distinct phylogenetic groups (Figure 2A,B). The analysis demonstrated an 8.35% nucleotide change between the proposed VII.2 groups, while a 1.86% and a 1.64% nucleotide change were determined internally between each group, suggesting there is a phylogenetic split in these two VII.2 groups.

Cleavage site (CS) sequence comparison showed no amino acid differences amongst the five isolates examined (^112^RRQKR/F^117^) (Table 2). Exploring predicted epitope sites in the F protein [32] showed no changes. In contrast, a comparison of HN epitope sites [33,34] showed that isolate OR367434 had a mutation from I^249^ → M^249^ within epitope 2, while isolate OR367439 had a mutation from D^346^ → K^346^. The relative roles these mutations play in either serological responses generated by vaccination or prior infection are unknown; however, the M^249^ is observed in other genotypes, so it would suggest that this mutation is tolerated within this epitope site.

### 3.3. Phylogenetic Analysis of Genotype XIII APMV-1 Isolates

**Figure 3:**
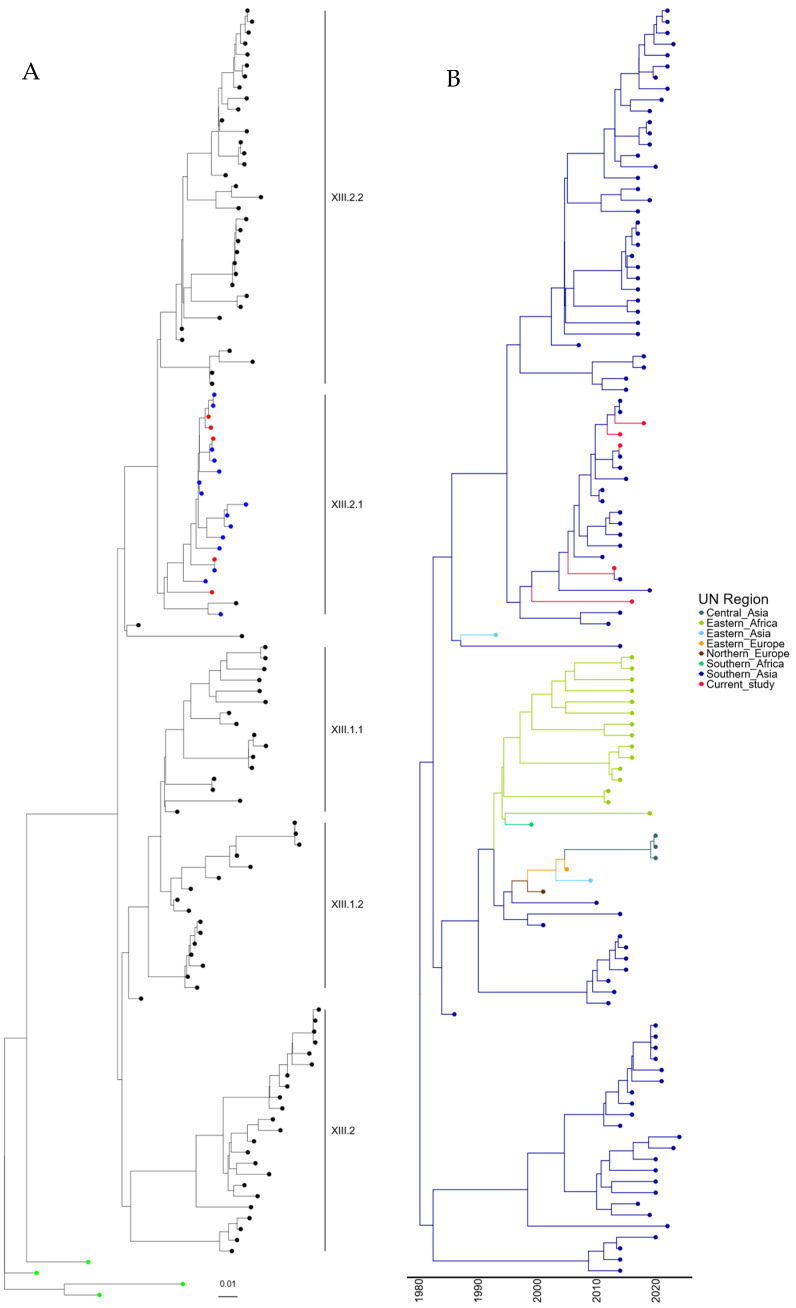
APMV-1 sequences designated genotype XIII are closely related to previous isolates observed in Pakistan. (**A**) Maximum-likelihood tree of the F-gene of five Pakistan isolates identified in this study, along with 109 F-gene sequences previously identified as genotype XIII. Isolates from this study are colored red, previous Pakistan samples are colored blue, root sequences are green, and remaining samples are colored black. (**B**) Estimation of time-scaled phylogenetic and mugration analysis of genotype VII.2 isolates. Estimation of time-scaled phylogenetic analysis was conducted on the M-L tree to determine the likely dates of the last common ancestors for each isolate; subsequently, mugration analysis was conducted to determine the likely incursion region. Regions are colored as stated.

To understand the mechanism of incursion of the five genotype XIII APMV-1 isolates identified, further phylogenetic analysis was conducted with the 109 genotype XIII sequences previously identified, along with the four recommended root sequences [23]. A maximum-likelihood tree was generated using these sequences and is shown in Figure 3A (and Figure S3). The five newly identified genotypes XIII are phylogenetically linked with those previously sequenced from Pakistan and are closely related to APMV-1 isolates previously designated as genotype XIII.2.1.

Again, to determine the time of the last common ancestor before the isolate was first detected in Pakistan, a time-scaled phylogenetic estimation analysis was undertaken. The first detection of genotype XIII.2.1 APMV-1 in Pakistan occurred in 2008, which is phylogenetically closely related to an outbreak in Iran (2011). However, the remaining Pakistan isolates (*n* = 18) are all phylogenetically linked, and the last common ancestor shared outside of Pakistan was 02/1993 (lower limit 05/1991, upper limit = 10/1995) (Figure 3B). Analysis of the region of incursion strongly suggests incursion from Southern Asia, where genotype XIII appears to have originated, with the first detection in 1982 (Figure 3B). It does, however, appear that genotypes XIII.2.1 and XIII.2.2 have remained almost exclusively in Southern Asia, whereas genotype XIII.1.1 and XIII.1.2 have been detected in central Asia, Eastern Africa, and both Eastern and Northern Europe (Figure 3B). This does, however, demonstrate that genotype XIII APMV-1 may have been circulating in Pakistan before the initial detection in 2007.

Cleavage site analysis (Table 2) demonstrated a single change in isolate OR367431 (Q^114^ → R^114^), while all remaining CS sequences remained unchanged. Analysis of F and HN epitopes showed conservation across all sites examined, with only isolate OR367437, which had a D^347^ → E^347^ mutation in epitope 3 of HN.

### 3.4. Phylogenetic Analysis of Genotype XXI APMV-1 Isolates

**Figure 4:**
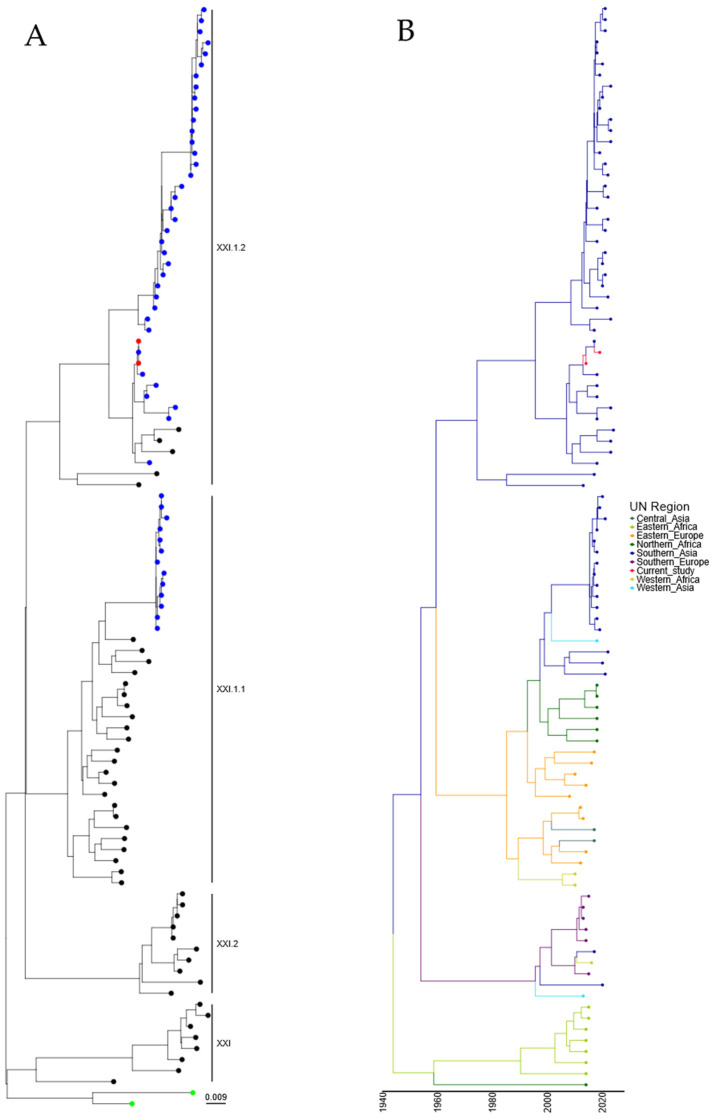
APMV-1 sequences designated genotype XXI are closely related to previous isolates observed in Pakistan. (**A**) Maximum-likelihood tree of the F-gene of five Pakistan isolates identified in this study, along with 97 F-gene sequences previously identified as genotype XXI. Isolates from this study are colored red, and previous Pakistan samples are colored blue, root sequences are green, and remaining samples are colored black. (**B**) Estimation of time-scaled phylogenetic and mugration analysis of genotype VII.2 isolates. Estimation of time-scaled phylogenetic analysis was conducted on the M-L tree to determine the likely dates of the last common ancestors for each isolate; subsequently, mugration analysis was conducted to determine the likely incursion region. Regions are colored as stated.

Following the identification of two genotype XXI isolates, further analysis was undertaken with a comparison of the two isolates characterized in this study with the ninety-seven genotype XXI isolate sequences available utilizing root sequences as described previously [23]. Maximum-likelihood phylogeny suggests that these isolates were genotype XXI.1.2, which was previously detected in Pakistan (Figure 4A and Figure S4). Genotype XXI isolates are commonly associated with pigeons, and the first full-length F-gene clone from genotype XXI was identified in 2005 (JF824032, Russia). Again, estimation of time-scaled phylogenetic analysis suggested that the last common ancestor before incursion into Southern Asia of genotype XXI.1.2 was 05/1971 (lower boundary 1961, upper boundary 1981), with this isolate next appearing in Eastern Europe (Figure 4B). However, the analysis of this tree shows theoretical common ancestors pre-dating the previously hypothesized date of PPMV-1 emergence in the Middle East (early-mid 1960s), with the first PPMV-1 sequence identified from an isolate from 1978 [36]. However, torticollis in pigeons was described as far back as 1927 [37], although there is no evidence that this is due to APMV-1/PPMV-1 infection. Cleavage site and epitope sequence analysis of the two genotype XXI.1.2 isolates demonstrated no mutations across any of the sites analyzed (Table 2).

## 4. Discussion

Avian paramyxovirus-1 (APMV-1) continues to cause economic losses across multiple locations in Pakistan. Understanding the genetic diversity of these viruses is critical to being able to drive forward mitigating activities and prevent the impact of incursions through preventative vaccination. Here, we describe thirteen newly sequenced complete genomes isolated from birds across Pakistan and have examined the phylogenetic relationship between these novel APMV-1 genomes with those previously described from Pakistan and geographically relevant countries over a temporal period to determine whether distinct routes of incursion have occurred. Continued analysis within regions where APMV-1 is endemic is essential to determine the presence of new, novel APMV-1 in the region and to understand the mechanisms of global spread. Given the extensive genetic diversity facilitated by a diverse range of susceptible avian species and the presence of highly mobile wild and migratory waterfowl acting as natural reservoirs, it is imperative for disease-endemic countries like Pakistan to continually monitor viral evolution and the molecular epidemiology of APMV-1 circulating nationwide.

APMV-1 strains representing genotypes II, VII, XIII, and XXI were previously detected in various hosts and geographic locations across Pakistan [14,15,17,20,38,39,40,41,42,43]. From our analysis, the detection of a single genotype II virus likely indicates the presence of the vaccine strain during sampling. Vaccine strains are generally non-virulent and should not appear in clinically diseased birds. However, the clinical signs of Newcastle disease can resemble those of other respiratory infections in birds, such as infectious bronchitis virus, infectious laryngotracheitis virus, and other respiratory pathogens. This similarity can lead clinicians to suspect NDV infection based on clinical symptoms alone. This challenge is further exacerbated by the fact that many veterinary laboratories in Pakistan are not equipped for antigen-based confirmatory testing, differential diagnosis is not commonly practiced, and clinicians tend to rely heavily on clinical diagnosis. This particular case was referred by a field veterinarian to our laboratory for the isolation and identification of NDV, which might explain the detection of a genotype II vaccine strain in a commercial farm with a history of ND vaccination. This situation underscores the importance of incorporating thorough clinical and antigen-based differential diagnostics for respiratory pathogens in future laboratory investigations and research studies. Notably, APMV-1 of genotype XIII was identified as the cause of poultry outbreaks [44] and was associated with mild respiratory infections in poultry workers [20]. Despite the proposal that genotype XIII APMV-1 had been replaced by genotype VII between 2010 and 2013 [40,44], we have determined an isolate from 2014 was present within Pakistan, which was genotype XIII, so this initial hypothesis was incorrect. These current findings underscore the presence of genotype XIII viruses in the field, indicating a potential need to revise diagnostic assays for detecting these circulating viruses.

Genotype XXI.1.2 isolates have been observed previously in Pakistan and Bangladesh, and the isolates studied here are closely related to previous isolates from Pakistan, again suggesting no novel incursion. However, the time-resolved analysis indicated that the time to the last common ancestor was before the current hypothesized date of introduction of APMV-1 into Columbiformes (mid-1960s). This discrepancy between isolation dates and predicted dates for most recent common ancestors is likely due to under-sampling. Indeed, the branch length between the different clades would suggest that intermediate PPMV-1 isolate sequences are not present. This under-sampling, especially during the initial spread of PPMV-1 (genotypes VI, XX, and XXI), has resulted in large confidence values on subsequent dates that do not fit the current known PPMV-1 worldwide outbreak. Although dating the common ancestors of genotype XXI isolates has proved difficult, we have shown that the two genotype XXI.1.2 isolates identified in this study are phylogenetically related to other Pakistani genotype XXI.1.2, again demonstrating that these were not the initial or novel incursions. However, unless sampling of historical PPMV-1 samples occurs, we may consistently see this inconclusive date for the time of common ancestors for this genotype.

All five isolates determined to be genotype VII.2 in this study were strongly associated with previous detections within Pakistan/Southern Asia, suggesting that these were not novel incursions. Interestingly, these Southern Asian isolates have spread from within this region to other parts of Asia, Eastern Africa, and into Europe and appear to be the root of the outbreak that occurred in Belgium/Netherlands/Luxembourg in 2018 [45], which demonstrates despite the high mortality consistently observed with this genotype, they can spread globally, and this is most likely through transit by human intervention and not through wild bird transmission. The classification system proposed by Dimitrov et al. [23] categorizes APMV-1 strains into different genotypes and sub-genotypes, including the establishment of a novel cluster. The sub-genotype classification was accomplished by phylogenetically analyzing the complete F gene for all strains. While all APMV-1 strains met the proposed criteria, the genotype VII.2 viruses isolated from Pakistan formed a distinct cluster to those previously described from the same genotype, with the latter being commonly detected in Southern Africa (Botswana, Mozambique, Namibia, and South Africa). Based on the analysis and identification of a nucleotide difference of 8.35% but a within-group nucleotide difference of 1.86% and 1.69%, we are proposing that these should now be identified as genotypes VII.2.1 and VII.2.2, based on the criteria defined by Dimitrov et al. [23]. The detection of divergent APMV-1s in Pakistan implies ongoing viral evolution and an epizootic nature transcending geographic boundaries. It may also reflect unsampled ancestry with significant gaps in our knowledge of the distribution and dissemination of these viruses. The significant role played by various avian species in disseminating the virus, especially in poultry production settings, is highlighted here, emphasizing the need for further understanding the potential role of these avian species in virus dissemination.

Vaccination against NDV has been practiced for decades, commonly utilizing avirulent APMV-1 (e.g., La Sota, Hitchner B1, Ulster 2C), although in regions where NDV is endemic, mesogenic strains (e.g., Muketshwar) are also used to boost immune responses [46]. However, with the rapidly evolving situation with APMV-1, it has been hypothesized that current vaccine strains are not able to protect against contemporary strains with vaccine breakthroughs observed [47,48,49], although studies do demonstrate that if suitable antibody titers are met, then current vaccines will protect but not completely inhibit shedding of virulent APMV-1 [25,50,51]. In Pakistan, vaccination against ND commonly occurs on days 1, 7, and 16–18 with a live vaccine and sometimes a combination of live (mostly genotype-II viruses) and killed vaccines (genotype VII viruses). Specifically, broiler chickens typically receive ND live vaccines at the hatchery (day 1), days 7–10, and days 18–21. Layer chickens, on the other hand, follow a more extensive schedule with ND live vaccines at hatchery (day 1), days 7–10, 4 weeks, 8 weeks, and 12 weeks, and ND inactivated vaccines at 16–18 weeks, especially before the start of the laying period. For gamebirds such as pigeons and pheasants, the vaccination schedule is highly variable and depends on the caretaker, reflecting differences in the level of attention and resources dedicated to their health management. Previously, genotype VII APMV-1 strains have been detected in vaccinated flocks [25,52,53]. These detections may facilitate further viral evolution, leading to the emergence of novel variants or escape mutants. This is important where infection of vaccinated flocks occurs, with vaccination regimes dependent on the poultry facility and the respective farm consultant.

These investigations highlight the significant role played by various avian species, especially those in captivity within poultry production settings in Pakistan, in the dissemination and evolution of these viruses. While current genetic diversity and evolutionary analyses reveal strong relationships among viruses from different avian species, including poultry, limited information is available regarding the potential role of these avian species in virus dissemination. The genetic diversity of APMV-1, characterized by synonymous and non-synonymous substitutions in coding gene residues, plays a crucial role in viral evolution and subsequent adaptation to a wide range of hosts [38]. Interestingly, recent studies on these viruses in Pakistan have assessed substitutions that have been observed in both the nucleotide and amino acid sequences of the F and HN genes, particularly in biologically and functionally significant motifs [13,17,18,39,42,54,55,56]. However, while such studies can demonstrate the frequency of changes, linking substitutions to functional adaptation remains problematic for these viruses. As such, only the observation of alterations to defined motifs can lead to conclusions about evolution and protein functionality.

## 5. Conclusions

This investigation of Avian paramyxovirus-1 (APMV-1) in Pakistan has revealed substantial genetic diversity and ongoing evolution, particularly within genotype VII. The categorization of strains into distinct genotypes underscores the intricate nature of the genetic evolution of these viruses. This study underscores the significance of various avian species, especially in poultry settings, in acting as a source of virus and highlights challenges for vaccination efforts with the possibility of escape mutant generation. Continuous monitoring is imperative for comprehending and addressing the dynamic nature of APMV-1 for effective disease control and management.

## Figures and Tables

**Table 1:** List of isolates examined in this study.

Isolation Year	Host	City/Region	Isolate Name	Sample-Type Collected	Accession Number	Genotype
2009	Chicken	Sheikhupura	AoAV-1/UVAS-Pak/Ck/2009	* Trachea, lung, spleen, and cecal tonsil	OR367431	XIII.2.1
2010	Chicken	Rawalpindi	AoAV-1/UVAS-Pak/Ck/2010	* Trachea, lung, spleen, proventriculus, and cecal tonsil	OR367436	XIII.2.1
2010	Parrot	Lahore	AoAV-1/UVAS-Pak/Parrot/2010	* Trachea and lung	OR367432	XIII.2.1
2011	Pigeon	Lahore	AoAV-1/UVAS-Pak/Pigeon/2011	* Trachea and brain	OR367435	XXI.1.2
2012	Chicken	Gujranwala	AoAV-1/UVAS-Pak/Ck/2012	* Trachea, lung, spleen, and cecal tonsil	OR367433	VII.2
2012	Chicken	Rawalpindi	AoAV-1/UVAS-Pak/Ck/2012	* Trachea, lung, spleen, proventriculus, and cecal tonsil	OR367437	XIII.2.1
2014	Chicken	Rawalpindi	AoAV-1/UVAS-Pak/Ck/2014	* Trachea, lung, spleen, proventriculus, and cecal tonsil	OR367438	XIII.2.1
2014	Pheasant	Lahore	AoAV-1/UVAS-Pak/Pheasant/2014	* Trachea, lung, spleen, and proventriculus	OR367434	VII.2
2015	Pheasant	Lahore	AoAV-1/UVAS-Pak/Pheasant/2015	* Trachea, lung, spleen, and proventriculus	OR367439	VII.2
2015	Duck	Mianwali	AoAV-1/UVAS-Pak/Anseriforme/2015	* Trachea, oropharyngeal, and cloacal swabs	OR367440	VII.2
2015	Duck	Mianwali	AoAV-1/UVAS-Pak/Anseriforme/2015	* Trachea, oropharyngeal and cloacal swabs	OR367441	VII.2
2016	Pigeon	Lahore	AoAV-1/UVAS-Pak/Pigeon/2016	Brain, * trachea, and lungs	OR367442	XXI.1.2
2021	Chicken	Lahore	AoAV-1/UVAS-Pak/LHR/Ck/2021	* Trachea, lung, spleen, and cecal tonsil	OR367430	II

* Sample type processed for isolation of virus in embryonated chicken eggs.

**Table 2:** Comparison of F-gene cleavage site sequences and F and HN epitope sites. Comparison of F-gene cleavage site sequences and both predicted F-gene and HN epitope sequences [32,33,34]. Alignments were carried out using MAFFT ver 7.453-1 [35].

Genotype	Sequence	F-Gene Cleavage Site	F-Gene Epitopes			HN Epitope Sites			
			Site 1	Site 2	Site 3	Epitope 1	Epitope 2	Epitope 3	Epitope 4
VII.2	HQ697254 (ref)	^112^ RRQKR/F117	^72^ DKEA75	L343	A378	^193^ LSGCRDHSH201	^242^ ATPLGCDILCSKVTE256	^345^ PDEQDYQIR353	D494 513RVTRVSSSS521
	OR367433	^112^ -----/-117	^72^ ----75	-343	-378	^193^ ---------201	^242^ ---------------256	^345^ ---------353	-494 513---------521
	OR367434	^112^ -----/-117	^72^ ----75	-343	-378	^193^ ---------201	^242^ -------M-------256	^345^ ---------353	-494 513---------521
	OR367439	^112^ -----/-117	^72^ ----75	-343	-378	^193^ ---------201	^242^ ---------------256	^345^ -K-------353	-494 513---------521
	OR367440	^112^ -----/-117	^72^ ----75	-343	-378	^193^ ---------201	^242^ ---------------256	^345^ ---------353	-494 513---------521
	OR367441	^112^ -----/-117	^72^ ----75	-343	-378	^193^ ---------201	^242^ ---------------256	^345^ ---------353	-494 513---------521
XIII.2.1	MH019282 (ref)	^112^ RRQKR/F117	^72^ DKEA75	L343	A378	^193^ LSGCRDHSH201	^242^ ATPLGCDMLCSKVTE256	^345^ PDDQDYQIQ353	D494 513RVTRVSSSS521
	OR367431	^112^ --R--/-117	^72^ ----75	-343	-378	^193^ ---------201	^242^ ---------------256	^345^ ---------353	-494 513---------521
	OR367432	^112^ -----/-117	^72^ ----75	-343	-378	^193^ ---------201	^242^ ---------------256	^345^ ---------353	-494 513---------521
	OR367436	^112^ -----/-117	^72^ ----75	-343	-378	^193^ ---------201	^242^ ---------------256	^345^ ---------353	-494 513---------521
	OR367437	^112^ -----/-117	^72^ ----75	-343	-378	^193^ ---------201	^242^ ---------------256	^345^ --E------353	-494 513---------521
	OR367438	^112^ -----/-117	^72^ ----75	-343	-378	^193^ ---------201	^242^ ---------------256	^345^ ---------353	-494 513---------521
XXI.1.2	KU885949 (ref)	^112^ RRQKR/F117	^72^ DKEA75	L343	A378	^193^ LSGCRDHSH201	^242^ ATPLGCDMLCSKVTE256	^345^ PDEQDYQIR353	D494 513RVTRVSSSS521
	OR367435	^112^ -----/-117	^72^ ----75	-343	-378	^193^ ---------201	^242^ ---------------256	^345^ ---------353	-494 513---------521
	OR367442	^112^ -----/-117	^72^ ----75	-343	-378	^193^ ---------201	^242^ ---------------256	^345^ ---------353	-494 513---------521

## Data Availability

Sequencing data available on NCBI.

## Supplementary Materials

The following supporting information can be downloaded at: https://www.mdpi.com/article/10.3390/v16091414/s1, Figure S1: Phylogenetic analysis of F-gene sequences from Pakistan isolates examined in this study. Figure S2: Phylogenetic analysis of APMV-1 sequences previously designated genotype VII.2 and closely related isolates observed in this study. Figure S3: Phylogenetic analysis of APMV-1 sequences previously designated genotype XIII and closely related isolates observed in this study. Figure S4: Phylogenetic analysis of APMV-1 sequences previously designated genotype XXI and closely related isolates observed in this study.
