# Supplementary material for: Genomic Diversity and Evolutionary Insights of Avian Paramyxovirus-1 in Avian Populations in Pakistan
Source: Viruses. 2024 Sep 5;16(9):1414. doi: 10.3390/v16091414 (PMC11437410; doi:10.3390/v16091414)
Supplement: Supplementary file 1 [file viruses-16-01414-s001.zip › Figure S3.pdf]

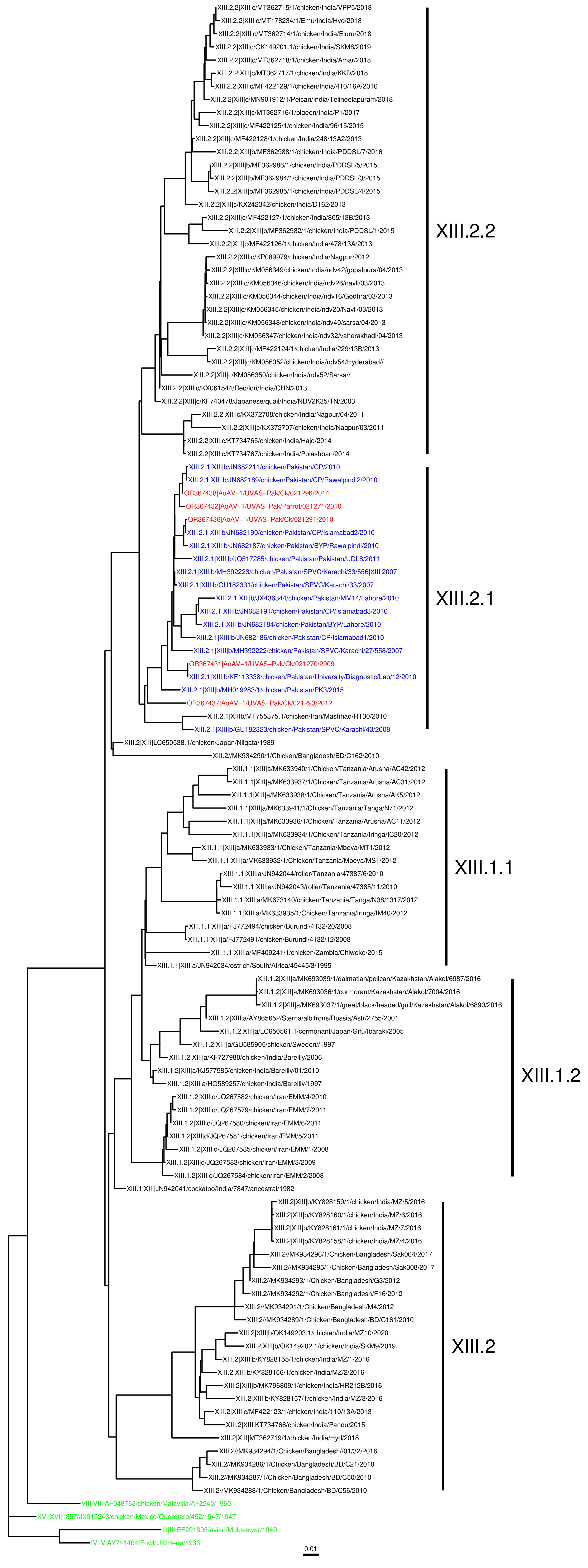

XIII.2.2|XIII|c/MT362715/1/chicken/India/VPP5/2018
XIII.2.2|XIII|c/MT178234/1/Emu/India/Hyd/2018
XIII.2.2|XIII|c/MT362714/1/chicken/India/Eluru/2018
XIII.2.2|XIII|c/OK149201.1/chicken/India/SKM8/2019
XIII.2.2|XIII|c/MT362718/1/chicken/India/Amar/2018
XIII.2.2|XIII|c/MT362717/1/chicken/India/KKD/2018
XIII.2.2|XIII|c/MF422129/1/chicken/India/410/16A/2016
XIII.2.2|XIII|c/MN901912/1/Peican/India/Telineelapuram/2018
XIII.2.2|XIII|c/MT362716/1/pigeon/India/P1/2017
XIII.2.2|XIII|c/MF422125/1/chicken/India/96/15/2015
XIII.2.2|XIII|c/MF422128/1/chicken/India/248/13A2/2013
XIII.2.2|XIII|b/MF362988/1/chicken/India/PDDSL/7/2016
XIII.2.2|XIII|b/MF362986/1/chicken/India/PDDSL/5/2015
XIII.2.2|XIII|b/MF362984/1/chicken/India/PDDSL/3/2015
XIII.2.2|XIII|b/MF362985/1/chicken/India/PDDSL/4/2015
XIII.2.2|XIII|c/KX242342/chicken/India/D162/2013
XIII.2.2|XIII|c/MF422127/1/chicken/India/805/13B/2013
XIII.2.2|XIII|b/MF362982/1/chicken/India/PDDSL/1/2015
XIII.2.2|XIII|c/MF422126/1/chicken/India/478/13A/2013
XIII.2.2|XIII|c/KP089979/chicken/India/Nagpur/2012
XIII.2.2|XIII|c/KM056349/chicken/India/ndv42/gopalpura/04/2013
XIII.2.2|XIII|c/KM056346/chicken/India/ndv26/navli/03/2013
XIII.2.2|XIII|c/KM056344/chicken/India/ndv16/Godhra/03/2013
XIII.2.2|XIII|c/KM056345/chicken/India/ndv20/Navli/03/2013
XIII.2.2|XIII|c/KM056348/chicken/India/ndv40/sarsa/04/2013
XIII.2.2|XIII|c/KM056347/chicken/India/ndv32/vaherakhadi/04/2013
XIII.2.2|XIII|c/MF422124/1/chicken/India/229/13B/2013
XIII.2.2|XIII|c/KM056352/chicken/India/ndv54/Hyderabad//
XIII.2.2|XIII|c/KM056350/chicken/India/ndv52/Sarsa//
XIII.2.2|XIII|c/KX061544/Red/lori/India/CHN/2013
XIII.2.2|XIII|c/KF740478/Japanese/quail/India/NDV2K35/TN/2003
XIII.2.2|XIII|c/KX372708/chicken/India/Nagpur/04/2011
XIII.2.2|XIII|c/KX372707/chicken/India/Nagpur/03/2011
XIII.2.2|XIII|c/KT734765/chicken/India/Hajo/2014
XIII.2.2|XIII|c/KT734767/chicken/India/Polashbari/2014
XIII.2.2
XIII.2.1|XIII|b/JN682211/chicken/Pakistan/CP/2010
XIII.2.1|XIII|b/JN682189/chicken/Pakistan/CP/Rawalpindi2/2010
OR367438|AoAV-1/UVAS-Pak/Ck/021296/2014
OR367432|AoAV-1/UVAS-Pak/Parrot/021271/2010
OR367436|AoAV-1/UVAS-Pak/Ck/021291/2010
XIII.2.1|XIII|b/JN682190/chicken/Pakistan/CP/Islamabad2/2010
XIII.2.1|XIII|b/JN682187/chicken/Pakistan/BYP/Rawalpindi/2010
XIII.2.1|XIII|b/JQ517285/chicken/Pakistan/Pakistan/UDL8/2011
XIII.2.1|XIII|b/MH392223/chicken/Pakistan/SPVC/Karachi/33/556|XIII|2007
XIII.2.1|XIII|b/GU182331/chicken/Pakistan/SPVC/Karachi/33/2007
XIII.2.1|XIII|b/JX436344/chicken/Pakistan/MM14/Lahore/2010
XIII.2.1|XIII|b/JN682191/chicken/Pakistan/CP/Islamabad3/2010
XIII.2.1|XIII|b/JN682184/chicken/Pakistan/BYP/Lahore/2010
XIII.2.1|XIII|b/JN682186/chicken/Pakistan/CP/Islamabad1/2010
XIII.2.1|XIII|b/MH392222/chicken/Pakistan/SPVC/Karachi/27/558/2007
OR367431|AoAV-1/UVAS-Pak/Ck/021270/2009
XIII.2.1|XIII|b/KF113338/chicken/Pakistan/University/Diagnostic/Lab/12/2010
XIII.2.1|XIII|b/MH019283/1/chicken/Pakistan/PK3/2015
OR367437|AoAV-1/UVAS-Pak/Ck/021293/2012
XIII.2.1|XIIIb/MT755375.1/chicken/Iran/Mashhad/RT30/2010
XIII.2.1|XIII|b/GU182323/chicken/Pakistan/SPVC/Karachi/43/2008
XIII.2.1
XIII.2|XIII|LC650538.1/chicken/Japan/Niigata/1989
XIII.2//MK934290/1/Chicken/Bangladesh/BD/C162/2010
XIII.1.1|XIII|a/MK633940/1/Chicken/Tanzania/Arusha/AC42/2012
XIII.1.1|XIII|a/MK633937/1/Chicken/Tanzania/Arusha/AC31/2012
XIII.1.1|XIII|a/MK633938/1/Chicken/Tanzania/Arusha/AK5/2012
XIII.1.1|XIII|a/MK633941/1/Chicken/Tanzania/Tanga/N71/2012
XIII.1.1|XIII|a/MK633936/1/Chicken/Tanzania/Arusha/AC11/2012
XIII.1.1|XIII|a/MK633934/1/Chicken/Tanzania/Iringa/IC20/2012
XIII.1.1|XIII|a/MK633933/1/Chicken/Tanzania/Mbeya/MT1/2012
XIII.1.1|XIII|a/MK633932/1/Chicken/Tanzania/Mbeya/MS1/2012
XIII.1.1|XIII|a/JN942044/roller/Tanzania/47387/6/2010
XIII.1.1|XIII|a/JN942043/roller/Tanzania/47385/11/2010
XIII.1.1|XIII|a/MK673140/chicken/Tanzania/Tanga/N38/1317/2012
XIII.1.1|XIII|a/MK633935/1/Chicken/Tanzania/Iringa/IM40/2012
XIII.1.1|XIII|a/FJ772494/chicken/Burundi/4132/20/2008
XIII.1.1|XIII|a/FJ772491/chicken/Burundi/4132/12/2008
XIII.1.1|XIII|a/MF409241/1/chicken/Zambia/Chiwoko/2015
XIII.1.1|XIII|a/JN942034/ostrich/South/Africa/45445/3/1995
XIII.1.1
XIII.1.2|XIII|a/MK693039/1/dalmatian/pelican/Kazakhstan/Alakol/6987/2016
XIII.1.2|XIII|a/MK693036/1/cormorant/Kazakhstan/Alakol/7004/2016
XIII.1.2|XIII|a/MK693037/1/great/black/headed/gull/Kazakhstan/Alakol/6890/2016
XIII.1.2|XIII|a/AY865652/Sterna/albifrons/Russia/Astr/2755/2001
XIII.1.2|XIII|a/LC650561.1/cormonant/Japan/Gifu/Ibaraki/2005
XIII.1.2|XIII|a/GU585905/chicken/Sweden//1997
XIII.1.2|XIII|a/KF727980/chicken/India/Bareilly/2006
XIII.1.2|XIII|a/KJ577585/chicken/India/Bareilly/01/2010
XIII.1.2|XIII|a/HQ589257/chicken/India/Bareilly/1997
XIII.1.2|XIII|d/JQ267582/chicken/Iran/EMM/4/2010
XIII.1.2|XIII|d/JQ267579/chicken/Iran/EMM/7/2011
XIII.1.2|XIII|d/JQ267580/chicken/Iran/EMM/6/2011
XIII.1.2|XIII|d/JQ267581/chicken/Iran/EMM/5/2011
XIII.1.2|XIII|d/JQ267585/chicken/Iran/EMM/1/2008
XIII.1.2|XIII|d/JQ267583/chicken/Iran/EMM/3/2009
XIII.1.2|XIII|d/JQ267584/chicken/Iran/EMM/2/2008
XIII.1.2
XIII.1|XIII|JN942041/cockatoo/India/7847/ancestral/1982
XIII.2|XIII|b/KY828159/1/chicken/India/MZ/5/2016
XIII.2|XIII|b/KY828160/1/chicken/India/MZ/6/2016
XIII.2|XIII|b/KY828161/1/chicken/India/MZ/7/2016
XIII.2|XIII|b/KY828158/1/chicken/India/MZ/4/2016
XIII.2//MK934296/1/Chicken/Bangladesh/Sak064/2017
XIII.2//MK934295/1/Chicken/Bangladesh/Sak008/2017
XIII.2//MK934293/1/Chicken/Bangladesh/G3/2012
XIII.2//MK934292/1/Chicken/Bangladesh/F16/2012
XIII.2//MK934291/1/Chicken/Bangladesh/M4/2012
XIII.2//MK934289/1/Chicken/Bangladesh/BD/C161/2010
XIII.2|XIII|b/OK149203.1/chicken/India/MZ10/2020
XIII.2|XIII|b/OK149202.1/chicken/India/SKM9/2019
XIII.2|XIII|b/KY828155/1/chicken/India/MZ/1/2016
XIII.2|XIII|b/KY828156/1/chicken/India/MZ/2/2016
XIII.2|XIII|b/MK796809/1/chicken/India/HR212B/2016
XIII.2|XIII|b/KY828157/1/chicken/India/MZ/3/2016
XIII.2|XIII|c/MF422123/1/chicken/India/110/13A/2013
XIII.2|XIII|KT734766/chicken/India/Pandu/2015
XIII.2|XIII|MT362719/1/chicken/India/Hyd/2018
XIII.2//MK934294/1/Chicken/Bangladesh//01/32/2016
XIII.2//MK934286/1/Chicken/Bangladesh/BD/C21/2010
XIII.2//MK934287/1/Chicken/Bangladesh/BD/C50/2010
XIII.2//MK934288/1/Chicken/Bangladesh/BD/C56/2010
XIII.2
VIII|VIII|AF048763/chicken/Malaysia/AF2240/1960
XVI|XVI|1857/JX915243/chicken/Mexico/Queretaro/452/1947/1947
III|III|EF201805/avian/Mukteswar/1940
IV|IV|AY741404/Fowl/UK/Herts/1933
0.01
