# Supplementary figures and images for: Genomic Diversity and Evolutionary Insights of Avian Paramyxovirus-1 in Avian Populations in Pakistan
Source: Viruses. 2024 Sep 5;16(9):1414. doi: 10.3390/v16091414 (PMC11437410; doi:10.3390/v16091414)

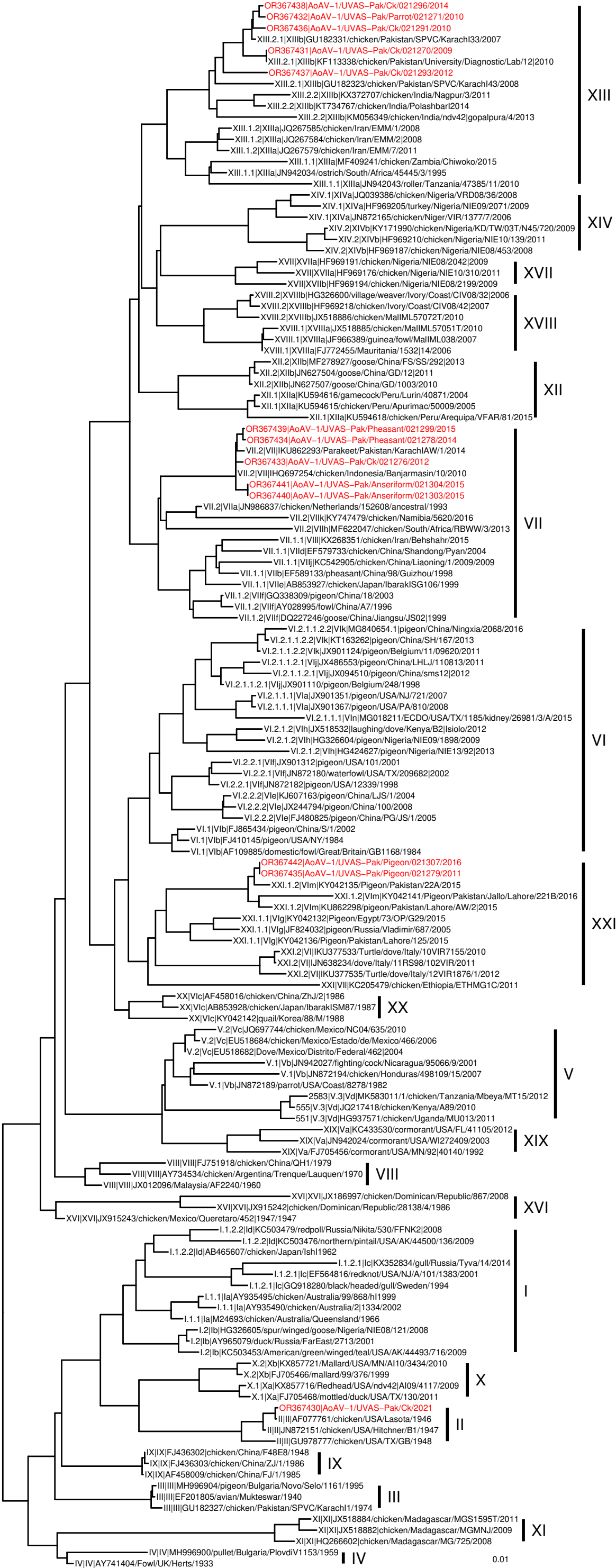

0.01

Supplement: Supplementary file 1 [file viruses-16-01414-s001.zip › Figure S1.pdf]

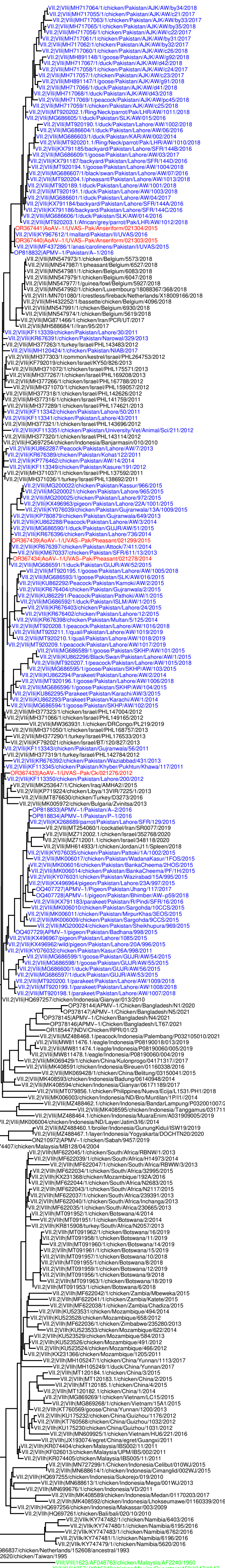

## VI.2.2

## VII.2.1

Supplement: Supplementary file 1 [file viruses-16-01414-s001.zip › Figure S2.pdf]

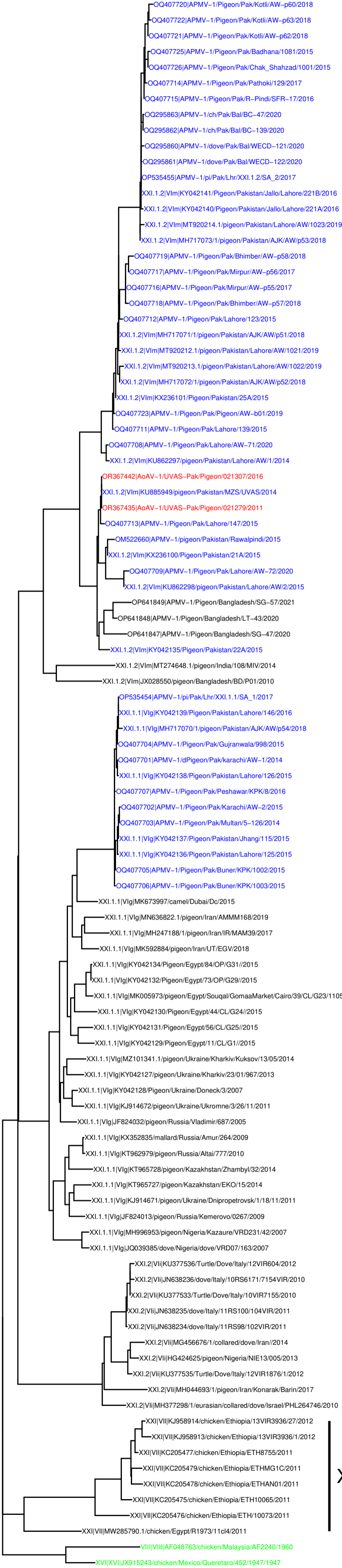

XXI.1.2

XXI.1.1

XXI.2

XXI

Supplement: Supplementary file 1 [file viruses-16-01414-s001.zip › Figure S4.pdf]
